# Effects of inappropriate empirical antibiotic therapy on mortality in patients with healthcare-associated methicillin-resistant *Staphylococcus aureus* bacteremia: a propensity-matched analysis

**DOI:** 10.1186/s12879-016-1650-8

**Published:** 2016-07-15

**Authors:** Young Kyung Yoon, Dae Won Park, Jang Wook Sohn, Hyo Youl Kim, Yeon-Sook Kim, Chang-Seop Lee, Mi Suk Lee, Seong-Yeol Ryu, Hee-Chang Jang, Young Ju Choi, Cheol-In Kang, Hee Jung Choi, Seung Soon Lee, Shin Woo Kim, Sang Il Kim, Eu Suk Kim, Jeong Yeon Kim, Kyung Sook Yang, Kyong Ran Peck, Min Ja Kim

**Affiliations:** Department of Internal Medicine, Korea University College of Medicine, Seoul, Republic of Korea; Department of Internal Medicine, Yonsei University Wonju College of Medicine, Won Ju, Republic of Korea; Department of Internal Medicine, Chungnam National University Hospital, Daejon, Republic of Korea; Department of Internal Medicine, Chonbuk National University Medical School, Jeonju, Republic of Korea; Department of Internal Medicine, Kyung Hee University School of Medicine, Seoul, Republic of Korea; Department of Internal Medicine, Keimyung University Dongsan Hospital, Daegu, Republic of Korea; Department of Internal Medicine, Chonnam National University Medical School, Gwangju, Republic of Korea; Department of Internal Medicine, National Cancer Center, Seoul, Republic of Korea; Department of Internal Medicine, Sungkyunkwan University School of Medicine, Seoul, Republic of Korea; Department of Internal Medicine, Ewha Women’s University School of Medicine, Seoul, Republic of Korea; Department of Internal Medicine, Hallym University Sacred Heart Hospital, Anyang, Republic of Korea; Department of Internal Medicine, Kyungpook National University Hospital, Daegu, Republic of Korea; Department of Internal Medicine, Catholic University of Korea, College of Medicine, Seoul, Republic of Korea; Department of Internal Medicine, Seoul National University Bundang Hospital, Seoul, Republic of Korea; Department of Internal Medicine, Samyook Medical Center, Seoul, Republic of Korea; Department of Biostatistics, Korea University College of Medicine, Seoul, Republic of Korea

**Keywords:** Methicillin-resistant *Staphylococcus aureus*, Bacteremia, Risk factors, Treatment outcome, Anti-bacterial agents

## Abstract

**Background:**

The purported value of empirical therapy to cover methicillin-resistant *Staphylococcus aureus* (MRSA) has been debated for decades. The purpose of this study was to evaluate the effects of inappropriate empirical antibiotic therapy on clinical outcomes in patients with healthcare-associated MRSA bacteremia (HA-MRSAB).

**Methods:**

A prospective, multicenter, observational study was conducted in 15 teaching hospitals in the Republic of Korea from February 2010 to July 2011. The study subjects included adult patients with HA-MRSAB. Covariate adjustment using the propensity score was performed to control for bias in treatment assignment. The predictors of in-hospital mortality were determined by multivariate logistic regression analyses.

**Results:**

In total, 345 patients with HA-MRSAB were analyzed. The overall in-hospital mortality rate was 33.0 %. Appropriate empirical antibiotic therapy was given to 154 (44.6 %) patients. The vancomycin minimum inhibitory concentrations of the MRSA isolates ranged from 0.5 to 2 mg/L by E-test. There was no significant difference in mortality between propensity-matched patient pairs receiving inappropriate or appropriate empirical antibiotics (odds ratio [OR] = 1.20; 95 % confidence interval [CI] = 0.71–2.03). Among patients with severe sepsis or septic shock, there was no significant difference in mortality between the treatment groups. In multivariate analyses, severe sepsis or septic shock (OR = 5.45; 95 % CI = 2.14–13.87), Charlson’s comorbidity index (per 1-point increment; OR = 1.52; 95 % CI = 1.27–1.83), and prior receipt of glycopeptides (OR = 3.24; 95 % CI = 1.08–9.67) were independent risk factors for mortality.

**Conclusion:**

Inappropriate empirical antibiotic therapy was not associated with clinical outcome in patients with HA-MRSAB. Prudent use of empirical glycopeptide therapy should be justified even in hospitals with high MRSA prevalence.

## Background

Methicillin-resistant *Staphylococcus aureus* (MRSA) has been a major cause of healthcare-associated bacteremia [1, 2]. MRSA bacteremia (MRSAB), with a mortality rate as high as 40 %, is a grave concern because a clinical cure may not be achieved using standard therapy in some cases [3–6]. Evidence-based therapy and the identification of mortality-related risk factors for MRSAB continue to represent significant clinical challenges for clinicians.

Previous studies determined the independent predictors of mortality among patients with MRSAB, including old age (≥60 years), underlying cardiac diseases, a higher Charlson’s comorbidity index, pneumonia, septic shock, metastatic infection, non-eradicable foci, and higher vancomycin minimum inhibitory concentrations (MICs) of the MRSA isolates [7–9]. However, studies evaluating the presence of relationships between clinical outcome and inappropriate empirical antibiotic therapies in patients with MRSAB have yielded conflicting results [10–16]. The conflicting results on the benefits of early empirical antibiotic therapy are probably due to differing definitions of “inappropriate” therapy on the basis of in vitro susceptibility data, impact of potentially confounding variables, and selection or information biases such as the baseline severity of illness [17, 18]. The definition and criteria items used to denote the appropriate antibiotic therapy should include the criterion matching the in vitro susceptibility and the timing of the administration of the antibiotics as well as their optimal dose and usage [19, 20]. A recent meta-analysis involving 510 MRSAB episodes demonstrated an overall 2-fold increased survival benefit with the administration of appropriate empirical therapy for MRSAB episodes, but there are some problems with heterogeneity across the studies [21]. In the meanwhile, the prudent use of glycopeptides has been an important component of hospital antimicrobial stewardship programs to contain the emergence of glycopeptide resistance, especially in high-prescribing countries with a high prevalence of MRSA. Thus, empirical glycopeptides use for MRSAB should be guided on the basis of scientific data.

The purpose of this study was to assess the influence of inappropriate empirical antimicrobial therapy on mortality in patients with healthcare associated-MRSAB (HA-MRSAB) in a hospital setting in which the prevalence of MRSA was high.

## Methods

### Study design and setting

A prospective, multi-center, observational study was conducted to compare clinical outcomes between patients receiving inappropriate or appropriate antibiotics for the treatment of HA-MRSAB in 15 teaching hospitals in the Republic of Korea from February 2010 to July 2011. This cross-sectional study was performed in accordance with the Strengthening the Reporting of Observational Studies in Epidemiology (STROBE) guidelines. Two analytical strategies were used: (1) for the non-matched case–control study (*n* = 345), the clinical outcomes of 154 patients who received appropriate empirical antibiotic treatment was compared with those of 191 patients who received inappropriate treatment; and (2) for the propensity score- matched (1:1) case–control study, the outcome was compared between 127 pairs of patients who received inappropriate or appropriate empirical antibiotics. Cases were defined as the patients treated with inappropriate empirical antibiotics, and controls were defined as those with HA-MRSAB who were treated with appropriate empirical antibiotics. The primary endpoint was all-cause in-hospital mortality. The secondary endpoints were mortality attributable to MRSAB, persistent fever, and persistent MRSAB.

All 15 participating hospitals have long had a high prevalence of MRSA, which accounted for about approximately 70 % of all *S. aureus* isolates. Clinical data were collected at each participating site via a standardized web-based case report form. A representative infectious disease specialist from each site prospectively reviewed and interpreted clinical data for the enrolled patients. A trough serum concentration of vancomycin was measured routinely in 13 of the 15 participating hospitals in this study.

### Study population

Study subjects included hospitalized adult patients (aged ≥18 years) with HA-MRSAB who had ≥1 positive blood culture for MRSA. Our study excluded the subjects who did not receive the active antibiotics against MRSA, after confirming that the index blood culture was positive for MRSA. All patients were followed up until death or hospital discharge. If a patient had more than 1 episode of HA-MRSAB during the study period, only the first episode was included. Patients with polymicrobial bacteremia were excluded from the analysis specifically evaluating the influence of empirical antimicrobial therapy for HA-MRSAB. Physicians treated patients according to routine medical practices without standardized protocols of intervention.

### Definitions

Patients with healthcare-associated or nosocomial MRSAB were included in this study. MRSAB diagnosed within 48 h of hospital admission was considered healthcare-associated infection, if the patient met any healthcare-associated criteria in the preceding 3 months, including admission to a hospital, nursing home, or other healthcare facility for more than 2 days, specialized home care, treatment at a medical day unit, surgery, dialysis, or permanent indwelling catheters. MRSAB diagnosed after 48 h of hospital admission was considered nosocomial infection.

The primary focus of HA-MRSAB was determined by the organ affected and classified as follows: catheter-related bloodstream infection (CR-BSI) [22], lower respiratory tract, intra-abdominal, urinary tract, skin and soft tissue, bone and joint, or central nervous system infection; or unknown when no other site of infection was evident [23]. Sepsis, severe sepsis, and septic shock were diagnosed in accordance with standard criteria [24].

Definitive therapy was defined as the antibiotic therapy administered subsequent to the receipt of the final blood culture and antibiotic susceptibility results [17]. Empirical antibiotic therapy was defined as initial antibiotic treatment started before the pathogen was identified and antimicrobial susceptibility test results were obtained. Empirical antibiotic therapy was defined as appropriate if the pathogen was shown in *in vitro* studies to be susceptible to the antibiotic administered intravenously with optimal dosing and if the timing of administration was within 48 h of obtaining the index positive blood culture. If it did not meet any of these criteria, it was regarded as inappropriate antibiotic therapy. Prior antibiotic exposure was defined as the receipt of >3 doses of antibiotics within 3 months before the occurrence of HA-MRSAB. The duration of bacteremia after definitive antibiotic therapy was calculated as the number of days from initiation of definitive antibiotic treatment against HA-MRSAB to the day on which the first negative blood culture was obtained.

Mortality attributable to MRSA bacteremia was defined as death with positive blood cultures for MRSA and persistent fever, but no other definitive causes of death. Persistent MRSAB and persistent fever were defined as positive blood cultures for MRSA for ≥7 days and as a fever ≥38.0 °C for ≥7 days after the commencement of appropriate antibiotic treatment, respectively.

### Variables

The clinical data of patients were extracted from medical records, including age, gender, medical history, comorbid medical conditions, Charlson’s comorbidity index [25], primary site of infection, risk factors predisposing to infections within 3 months before the occurrence of HA-MRSAB, APACHE II scores [26], Pitt’s bacteremia score [27] on the day of index positive blood culture sampling, presence of severe sepsis or septic shock, mortality attributable to MRSAB, and in-hospital mortality.

### Microbiological tests

The identification of MRSA and antibiotic susceptibility tests were performed in each hospital using the MicroScan Pos Combo Panel Type 6 automated system (Baxter Diagnostics, West Sacramento, CA, USA) or VITEK II automated system (bioMérieux, Hazelwood, MO, USA). All MRSA isolates from index blood cultures were collected at the coordinating center and immediately stored at −70 °C until August 2012 when the MICs of vancomycin were further determined by the E-test (bioMérieux, Marcy l’Etoile, France) according to the manufacturer’s instructions.

### Statistical analysis

Categorical variables were denoted as count (proportion) and compared using Pearson’s chi-squared test, Fisher’s exact test, or McNemar’s test. Continuous variables were expressed as the mean ± standard deviation or median (inter-quartile range [IQR]) and compared using two-sample Student’s *t*-tests, the Mann–Whitney U test, or Wilcoxon’s signed-rank test. All tests were two-tailed, and a *P*-value <0.05 was considered statistically significant.

For the covariate adjustment between patients receiving inappropriate or appropriate empirical antibiotic therapy, variables that represented the probability of being treated inadequately were found. In univariate analysis of 345 patients with HA-MRSAB, baseline and clinical characteristics were compared between the two treatment groups. Variables at the 10 % significance level were considered independent variables for the propensity scores, including category of infection, trauma, primary focus of HA-MRSAB (such as CR-BSI, pneumonia, or urinary tract infection), severe sepsis or septic shock, infective endocarditis, retention of foreign body, surgery, and recent exposure to immunosuppressive agents such as systemic corticosteroids on a regular basis or antineoplastic chemotherapy, third-generation cephalosporins, or fluoroquinolones during the preceding 30 days. Patients who received inappropriate empirical antibiotic therapy were matched 1:1 with patients who received appropriate empirical antibiotic therapy with regard to 5 digits of the propensity score without replacement [28, 29]. To confirm whether we had a good match, the absolute standardized differences for each covariate in the model and distribution of propensity scores were evaluated [30]. To determine the predictors of in-hospital mortality in patients with HA-MRSAB, multivariate conditional logistic regression analyses within the matched data set were performed with all variables with a *P*-value ≤0.10 in the univariate analysis as well as the main variable of inappropriate empirical antibiotic therapy.

Within the unmatched data set, inverse probability of treatment weighting (IPTW) based on the propensity score was also used to control for bias in treatment assignment [30, 31].

Data were analyzed using IBM SPSS Statistics version 20.0 (IBM Corporation, Armonk, NY, USA), R 2.15.2 (The R Foundation for Statistical Computing, Vienna, Austria), and SAS 9.2 (SAS Institute, Cary, NC, USA).

## Results

During the study period, a total of 345 patients with HA-MRSAB were included in our analysis. Their demographic and clinical characteristics are presented in Table 1. The most common primary focus of HA-MRSAB was CR-BSI (51.3 %), followed by pneumonia (11.6 %), surgical wound infection (7.0 %), intra-abdominal infection (21.0 %), skin and soft tissue infection (4.9 %), and others (4.2 %). The all-cause in-hospital mortality and MRSAB-related mortality rates were 33.0 (114/345) and 16.5 % (57/345), respectively. An in-hospital mortality rate of at least 25 % was noted for pneumonia (55 %), central nervous system infections, MRSAB of unknown origin (44.4 %), intra-abdominal infections (38.1 %), CR-BSI (33.3 %), and cardiovascular infections (28.6 %), whereas mortality rates of less than 25 % were observed for urinary tract infections, surgical wound infections, skin and soft tissue infections, bone and joint infections, and head and neck infections. Six patients died before the culture results were reported as follows: 4 patients presented with septic shock and 2 patients displayed high APACHE II scores of 30 and 39, respectively.

**Table 1:** Demographic and clinical characteristics of 345 patients with healthcare-associated methicillin-resistant *Staphylococcus aureus* bacteremia according to the appropriateness of initial empirical antimicrobial therapy

Variables	All (*n* = 345)	Appropriate (*n* = 154, 44.6 %)	Inappropriate (*n* = 191, 55.4 %)	Odds ratio (95 % confidence interval)
Male sex	154 (44.6)	97 (43.9)	124 (56.1)	0.92 (0.59–1.43)
Age (years), median (IQR)	67 (52–75)	66 (51–74)	67 (53–75)	0.99 (0.98–1.01)
Category of infection				
Healthcare-associated^a^	51 (14.8)	29 (18.8)	22 (11.5)	1.78 (0.98–3.25)
Nosocomial	294 (85.2)	125 (81.2)	169 (88.5)	
Comorbidity				
Malignancy	97 (28.1)	47 (30.5)	50 (26.2)	0.81 (0.50–1.29)
Metabolic	122 (35.4)	52 (33.8)	70 (36.6)	1.14 (0.73–1.77)
Trauma	29 (8.4)	10 (6.5)	19 (9.9)	1.59 (0.72–3.53)
Charlson’s comorbidity index^b^, median (IQR)	2 (1–4)	2 (1–5)	2 (1–4)	1.09 (1.00–1.20)
Primary focus of HA-MRSAB				
CR-BSI	177 (51.3)	93 (60.4)	84 (44.0)	0.52 (0.34–0.79)
Pneumonia	40 (11.6)	13 (8.4)	27 (14.1)	1.79 (0.89–3.59)
Clinical severity				
Development of severe sepsis or septic shock	99 (28.7)	50 (32.5)	49 (25.7)	0.72 (0.45–1.15)
Pitt’s bacteremia score at onset of bacteremia^c^, median (IQR)	1 (0–2)	1 (0–3)	1 (0–2)	1.08 (0.96–1.21)
Predisposing factors				
Surgical operation	84 (24.3)	29 (18.8)	55 (28.8)	1.74 (1.05–2.91)
Foreign body retention	15 (4.3)	12 (7.8)	3 (1.6)	0.189 (0.05–0.69)
Prior antibiotics use	219 (63.5)	99 (64.3)	120 (62.8)	0.94 (0.60–1.46)
Vancomycin MIC, mg/L	1 (1–1.5)	1 (1–1.5)	1 (1–1.5)	1.57 (0.81–3.04)
Outcomes				
In-hospital mortality	114 (33.0)	51 (33.1)	63 (33.0)	0.99 (0.63–1.56)
MRSAB-related mortality	57 (16.5)	24 (15.6)	33 (17.3)	1.13 (0.64–2.01)

*IQR* interquartile range, *APACHE* acute physiology and chronic health evaluation, *CR-BSI* catheter-related bloodstream infection, *MIC* minimum inhibitory concentration, *MRSAB* methicillin-resistant *Staphylococcus aureus* bacteremia

^a^MRSAB diagnosed within 48 h of hospital admission was considered healthcare-associated infection, if the patient presented with any healthcare-associated factor in the preceding 3 months

^b^Charlson’s comorbidity score was calculated at the onset of MRSA bacteremia infection

^c^Pitt’s bacteremia score and APACHE II scores were assessed at the onset of MRSA bacteremia

Of the 345 patients with HA-MRSAB, 154 (44.6 %) patients received appropriate empirical antibiotic therapy. The antibiotics used for definitive therapy were vancomycin (*n* = 201, 58.3 %), teicoplanin (*n* = 96, 27.8 %), arbekacin (*n* = 63, 18.3 %), linezolid (*n* = 34, 9.9 %), and tigecycline (*n* = 8, 2.3 %). Combination antibiotic therapy was administered to 15 patients as follows: glycopeptides plus rifampin (*n* = 11), linezolid plus arbekacin (*n* = 2), tigecycline plus arbekacin (*n* = 1), and teicoplanin plus arbekacin (*n* = 1).

For the non-matched case–control study (*n* = 345), the clinical outcomes of 154 patients who received appropriate empirical antibiotic therapy was compared with those of 191 patients who received inappropriate therapy. There were no significant differences in in-hospital mortality (odds ratio [OR] = 0.99; 95 % confidence interval [CI] = 0.63–1.56) or MRSAB-related mortality (OR = 1.13; 95 % CI = 0.64–2.01) between the two treatment groups (Table 1).

### Propensity score-matched analysis

For the propensity score-matched (1:1) case–control study, only 127 patient pairs were matched according to the propensity score, because some variables, namely catheter-related bloodstream infection, retention of foreign body, surgical operation, and Charlson’s comorbidity index, showed large differences between the two groups. Comparisons of demographic, clinical, and microbiological characteristics between the propensity-matched 127 pairs receiving inappropriate or appropriate empirical antibiotics are shown in Table 2. There were no significant differences in in-hospital mortality (OR = 1.20; 95 % CI = 0.71–2.03) or MRSAB-related mortality (OR = 1.34; 95 % CI = 0.68–2.62) between the two treatment groups (Table 2). The antibiotics used for the appropriate empirical antibiotic therapy were vancomycin (*n* = 93, 73.2 %), teicoplanin (*n* = 22, 17.3 %), and arbekacin (*n* = 12, 9.4 %).

**Table 2:** Comparison of clinical and microbiological characteristics and outcomes among patients with healthcare-associated methicillin-resistant *Staphylococcus aureus* bacteremia according to the appropriateness of empirical antibiotic therapy or treatment outcome in the propensity-matched analyses

Variables	Total	Empirical antibiotic therapy			Treatment outcome		
		Inappropriate (*n* = 127)	Appropriate (*n* = 127)	OR (95 % CI)	Non-survival (*n* = 81, 31.9 %)	Survival (*n* = 173, 68.1 %)	OR (95 % CI)
Male sex	165 (65.0)	84 (66.1)	81 (63.8)	1.03 (0.62–1.72)	59 (70.2)	106 (62.4)	0.66 (0.38–1.15)
Age (years), median (IQR)	67 (52–75)	68 (53–76)	65 (51–73)	0.99 (0.97–1.00)	72 (60–79)	63 (48–72)	1.03 (1.01–1.05)
Category of infection							
Healthcare-associated^a^	32 (12.6)	16 (12.6)	16 (12.6)	1.07 (0.52–2.23)	4 (4.8)	28 (16.5)	2.31 (0.91–5.84)
Nosocomial	222 (87.4)	111 (87.4)	111 (87.4)		80 (95.2)	142 (83.5)	
Comorbidity							
Malignancy	68 (26.8)	30 (23.6)	38 (29.9)	0.66 (0.37–1.16)	33 (39.3)	35 (20.6)	2.63 (1.46–4.73)
Trauma	24 (9.4)	14 (11.0)	10 (7.9)	1.33 (0.56–3.17)	7 (8.3)	17 (10.0)	0.93 (0.37–2.35)
Charlson’s comorbidity index^b^, median (IQR)	2 (1–4)	2 (1–4)	2 (1–4)	1.08 (0.97–1.19)	3 (2–6)	2 (0–2)	1.37 (1.21–1.54)
Predisposing factors							
Foreign body retention	4 (1.6)	2 (1.6)	2 (1.6)	1.00 (0.14–7.21)	1 (1.2)	3 (1.8)	0.71 (0.07–6.92)
Surgical operation	48 (18.9)	24 (18.9)	24 (18.9)	1.00 (0.53–1.87)	14 (16.7)	34 (20.0)	1.09 (0.56–2.12)
Prior antibiotic use	151 (59.4)	73 (57.5)	78 (61.4)	1.00 (0.60–1.66)	58 (69.0)	93 (54.7)	2.39 (1.33–4.30)
Third-generation cephalosporins	78 (30.7)	40 (31.5)	38 (29.9)	1.15 (0.68–1.95)	27 (32.1)	51 (30.0)	1.40 (0.81–2.44)
Fluoroquinolones	39 (15.4)	17 (113.4)	22 (17.3)	0.89 (0.45–1.75)	16 (19.8)	23 (13.5)	1.96 (0.99–3.91)
Glycopeptides	36 (14.2)	14 (11.0)	22 (17.3)	0.77 (0.38–1.57)	19 (22.6)	17 (10.0)	2.15 (1.05–4.41)
Primary focus of HA-MRSAB							
CR-BSI	144 (56.7)	72 (56.7)	72 (56.7)	1.03 (0.63–1.70)	52 (61.9)	92 (54.1)	1.03 (0.61–1.75)
Pneumonia	22 (8.7)	12 (9.4)	10 (7.9)	0.41 (0.62-3.21)	11 (13.1)	11 (6.5)	3.35 (1.46–7.67)
Clinical severity							
Development of severe sepsis or septic shock	75 (29.5)	33 (26.0)	42 (33.1)	0.64 (0.37–1.09)	43 (51.2)	32 (18.8)	5.24 (2.93–9.38)
Pitt’s bacteremia score^c^, median (IQR)	1 (0–3)	1 (0–3)	1 (0–3)	1.08 (0.94–1.24)	3 (1–4)	1 (0–2)	0..96 (0.82–1.12)
Vancomycin MIC, mg/L							
MIC ≥1.0 mg/L	211 (96.3)	106 (98.1)	105 (94.6)	1.15 (0.52–2.54)	69 (95.8)	142 (96.6)	1.76 (0.36–8.71)
MIC ≥1.5 mg/L	94 (42.9)	39 (36.1)	55 (49.5)	0.60 (0.31–1.16)	34 (47.2)	60 (40.8)	1.60 (0.91–2.83)
MIC ≥2 mg/L	19 (8.7)	8 (7.4)	11 (9.9)	0.47 (0.08–2.61)	9 (12.5)	10 (6.8)	2.46 (0.95–6.34)
Appropriate empirical antibiotic therapy, n (%)	127 (50.0)				42 (50.0)	85 (50.0)	1.20 (0.71–2.03)
Definitive therapy							
Vancomycin	145 (57.1)	68 (53.5)	77 (60.6)	0.75 (0.46–1.23)	48 (57.1)	97 (57.1)	1.00 (0.59–1.70)
Teicoplanin	75 (29.5)	38 (29.9)	37 (29.1)	1.04 (0.61–1.78)	26 (31.0)	49 (28.8)	1.11 (0.63–1.96)
Linezolid	28 (11.0)	16 (12.6)	12 (9.4)	1.38 (0.63–3.05)	9 (10.7)	19 (11.2)	0.95 (0.41–2.21)
Arbekacin	45 (17.7)	25 (19.7)	20 (15.7)	1.31 (0.69–2.51)	10 (11.9)	35 (20.6)	0.52 (0.24–1.11)
Tigecycline	4 (1.6)	2 (1.6)	2 (1.6)	1.00 (0.14–6.99)	2 (2.4)	2 (1.2)	2.05 (0.28–14.80)
Rifampin^d^	9 (3.5)	7 (5.5)	2 (1.6)	3.65 (0.74–17.9)	0	9 (5.3)	0.95 (0.91–0.98)
Outcomes							
In-hospital mortality	84 (33.1)	42 (33.1)	42 (33.1)	1.20 (0.71–2.03)			1.20 (0.71–2.03)
MRSAB-related mortality	40 (15.7)	21 (16.5)	19 (15.0)	1.34 (0.68–2.62)	40 (47.6)	0	2.03 (1.62–2.53)

*CR-BSI* catheter-related bloodstream infection, *IQR* interquartile range, *MIC* minimum inhibitory concentration, *MRSA* methicillin-resistant *Staphylococcus aureus*, *MRSAB* methicillin-resistant *Staphylococcus aureus* bacteremia

^a^MRSAB diagnosed within 48 h of hospital admission was considered healthcare-associated infection, if the patient presented with healthcare-associated factor in the preceding 3 months

^b^Charlson’s comorbidity index was calculated at the first identification of MRSA bloodstream infection

^c^Pitt’s bacteremia score was assessed at the first identification of MRSA bloodstream infection

^d^Rifampin, which was prescribed in the survivals for the maintenance combination treatment during the finishing step for the small number of study cases

To assess the effect of empirical antibiotic therapy according to the clinical severity of HA-MRSAB, the clinical outcomes of patients with HA-MRSAB in association with empirical antibiotic therapy and the severity of HA-MRSAB were analyzed (Table 3). For the subgroup with severe sepsis (*n* = 75), septic shock (*n* = 45), or a higher Charlson’s comorbidity index (≥3) (*n* = 108), there were no significant differences in either in-hospital mortality or MRSAB-related mortality between the patients receiving inappropriate or appropriate empirical antibiotic treatment (Table 3).

**Table 3:** Comparison of in-hospital mortality rates according to the clinical severity of healthcare-associated methicillin-resistant *Staphylococcus aureus* bacteremia in the propensity-matched analyses

	Empirical antibiotic therapy	In-hospital mortality, n (%)	OR (95 % CI)	MRSA-related mortality, n (%)	OR (95 % CI)
Severe sepsis or septic shock (*n* = 75)					
Yes	Appropriate	24 (57.1)	1.02 (0.41–2.56)	13 (31.0)	1.12 (0.42–2.96)
	Inappropriate	19 (57.9)		11 (33.3)	
No	Appropriate	18 (21.2)	1.21 (0.60–2.43)	6 (7.1)	1.57 (0.54–4.51)
	Inappropriate	23 (24.5)		10 (10.6)	
Septic shock (*n* = 45)					
Yes	Appropriate	20 (66.7)	1.00 (0.27–3.72)	12 (40.0)	1.00 (0.28–3.54)
	Inappropriate	10 (66.7)		6 (40.0)	
No	Appropriate	22 (22.7)	1.36 (0.73–2.55)	7 (7.2)	1.99 (0.78–5.10)
	Inappropriate31	32 (28.6)		15 (13.4)	
Charlson’s comorbidity index ≥3 (*n* = 108)					
Yes	Appropriate	31 (56.4)	0.62 (0.29–1.32)	13 (23.6)	0.83 (0.33–2.05)
	Inappropriate	24 (44.4)		11 (20.8)	
No	Appropriate	11 (15.3)	1.82 (0.79–4.18)	6 (8.3)	1.75 (0.60–5.09)
	Inappropriate	18 (24.7)		10 (13.7)	

*MRSA* methicillin-resistant *Staphylococcus aureus*

**Fig. 1:**
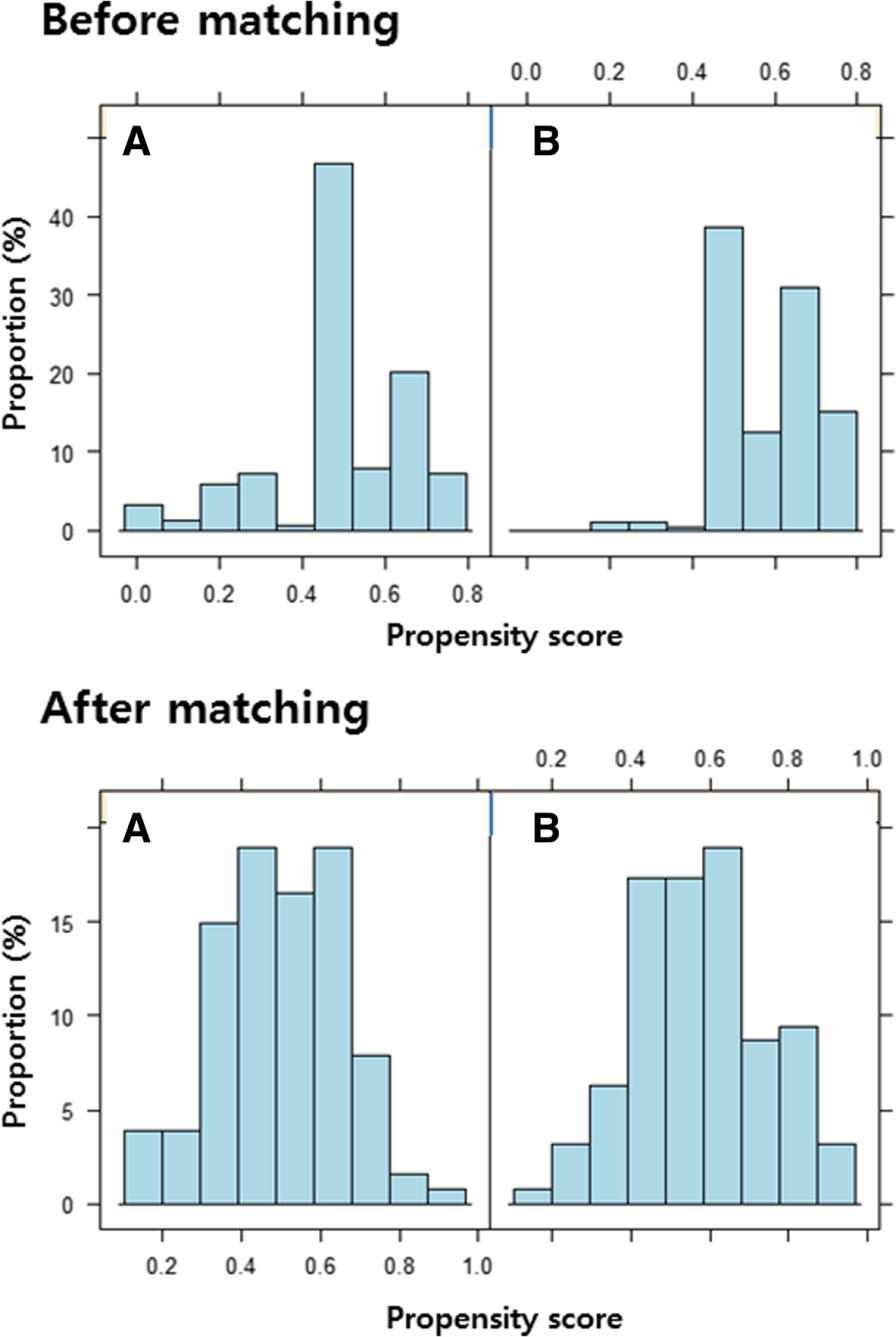
The distribution of propensity scores in unmatched and matched data sets for the appropriate empirical antibiotic treatment (**a**) and inappropriate empirical antibiotic treatment groups (**b**)

The covariate balance of independent variables for the propensity scores between the unmatched and matched data sets is presented in Table 4. In the propensity score-matched analysis, matched standardized differences should be less than 0.20 and preferably 0.10 for the balanced distribution of the relevant variables between the two groups [32, 33]. Figure 1 demonstrates graphic overlays of distribution of the propensity scores before and after matching between the two treatment groups.

**Table 4:** Covariate balance of independent variables for the propensity scores between unmatched and matched data sets

	Absolute standardized difference (%)	
	Unmatched data	Matched data
Nosocomial infection	20.49	0.00
Trauma	12.60	10.78
Surgical operation	23.56	0.00
Immunosuppressive agents	15.43	10.73
Prior exposure to third-generation cephalosporins	11.91	3.41
Prior exposure to fluoroquinolones	12.36	10.94
Prior exposure to glycopeptides	14.06	18.14
CR-BSI	33.30	0.00
Pneumonia	18.07	5.60
Urinary tract infection	19.90	18.11
Severe sepsis or septic shock	15.05	15.58
Retention of foreign body	29.78	0.00
Infective endocarditis	15.25	18.11
Charlson’s comorbidity index	21.35	16.12
Pitt’s bacteremia score	13.35	11.08
Age	10.47	13.89
Vancomycin MICs	15.67	17.59

*CR-BSI* catheter-related bloodstream infection, *MIC* minimum inhibitory concentration

The factors associated with all-cause in-hospital mortality in patients with HA-MRSAB were determined in the propensity score-matched set. For the 254 patients included, demographic, clinical, and microbiological characteristics were compared between 84 non-survivors and 170 survivors (Table 2).

In multivariate conditional logistic regression analysis, severe sepsis or septic shock (OR = 5.45; 95 % CI = 2.14–13.87), Charlson’s comorbidity index (per 1-point increment; OR = 1.52; 95 % CI = 1.27–1.83), and prior receipt of glycopeptides (OR = 3.24; 95 % CI = 1.08–9.67) were found to be independent risk factors for in-hospital mortality (Table 5).

**Table 5:** Multivariate conditional logistic regression analysis of risk factors for in-hospital mortality in patients with healthcare-associated methicillin-resistant *Staphylococcus aureus* bacteremia in the propensity-matched analyses

	Unmatched data set (*n* = 345)		Unmatched data set (IPTW, *n* = 345)		Matched data set (*n* = 254)	
Independent variable	OR (95 % CI)	*P*-value	OR (95 % CI)	*P*-value	OR (95 % CI)	*P*-value
Inappropriate empirical antibiotic therapy	1.26 (0.64–2.48)	0.499	1.33 (0.83–2.12)	0.236	NA^a^	0.988
Nosocomial infection	2.24 (0.87–5.80)	0.096	2.62 (1.28–5.37)	0.009		
Trauma	0.60 (0.18–1.97)	0.398	0.66 (0.28–1.52)	0.329	1.95 (0.50–7.56)	0.335
Surgical operation	1.20 (0.54–2.66)	0.656	1.22 (0.69–2.17)	0.493		
Prior exposure to third- generation cephalosporins	1.34 (0.68–2.63)	0.399	1.23 (0.76–2.00)	0.407	0.93 (0.41–2.10)	0.853
Prior exposure to fluoroquinolones	0.51 (0.22–1.22)	0.129	0.66 (0.35–1.24)	0.193	1.24 (0.44–3.51)	0.683
Prior exposure to glycopeptides	2.85 (1.20–6.77)	0.018	2.86 (1.55–5.28)	0.001	3.24 (1.08–9.67)	0.035
CR-BSI	0.92 (0.44–1.92)	0.825	0.79 (0.47–1.32)	0.360		
Pneumonia	0.37 (0.14–1.00)	0.050	0.28 (0.14–0.59)	0.001	2.15 (0.51–9.04)	0.296
Severe sepsis or septic shock	3.84 (1.85–7.96)	<0.001	3.64 (2.17–6.10)	<0.001	5.45 (2.14–13.87)	<0.001
Retention of foreign body	1.63 (0.40–6.71)	0.500	1.96 (0.62–6.18)	0.250		
Charlson’s comorbidity index (per 1-point increment)	0.66 (0.57–0.77)	<0.001	0.68 (0.61–0.75)	<0.001	1.52 (1.27–1.83)	<0.001
Pitt’s bacteremia score (per 1-point increment)	0.77 (0.64–0.93)	<0.001	0.76 (0.66–0.87)	<0.001	1.23 (0.99–1.54)	0.067
Age (per 1-year increment)	0.96 (0.94–0.98)	0.001	0.97 (0.95–0.98)	<0.001	1.02 (1.00–1.05)	0.105
Vancomycin MICs	0.48 (0.20–1.16)	0.103	0.42 (0.23–0.79)	0.007	1.55 (0.52–4.62)	0.433

*CR-BSI* catheter-related bloodstream infection, *IPTW* inverse probability of treatment weighted, *OR* odds ratio, *95 % CI* 95 % confidence interval, *MIC* minimum inhibitory concentration, *NA* not available

^a^These variables were not available because they displayed perfect marginal homogeneity with respect to each category concerning inappropriate initial empirical antibiotic therapy

As shown in Table 6, in multivariate logistic regression models of risk factors for in-hospital mortality using naïve, propensity score matching, or IPTW approaches, inappropriate empirical antibiotic therapy was not an independent predictor for mortality (Table 6).

**Table 6:** Evaluation of the association between inappropriate empirical antibiotic therapy and in-hospital mortality in patients with healthcare-associated methicillin-resistant *Staphylococcus aureus* bacteremia

		Number	*P*-value	OR	95 % CI	
					LL	UL
Model 1	Unmatched sample, no adjustment	345	0.979	1.01	0.64	1.58
Model 2	Unmatched sample with no PS, adjusted for all covariates	345	0.499	0.79	0.40	1.56
Model 3	Unmatched sample with IPTW, adjusted for all covariates	345	0.236	0.75	0.47	1.20
Model 4	PS-matched sample, no adjustment	254	0.984		NA^a^	.
Model 5	PS-matched sample adjusted for all covariates	254	0.988		NA^a^	.

*PS* propensity score, *IPTW* inverse probability of treatment weighted, *OR* odds ratio, *95 % CI* 95 % confidence interval, *LL* lower limit, *UL* upper limit

^a^These variables were not available because they displayed perfect marginal homogeneity with respect to each category concerning inappropriate initial empirical antibiotic therapy

### Microbiological factors and clinical outcomes

A total of 296 (85.8 %) MRSA isolates were available for analyzing vancomycin MICs using the E- test. The measured vancomycin MIC range was 0.5–2 mg/L; vancomycin MIC_50_ and MIC_90_ were both 1.5 mg/L. There was no difference in mortality outcomes between the two groups categorized as each reference cut-off of vancomycin MICs: ≥1.0 mg/L (OR = 1.76; 95 % CI = 0.36–8.71), ≥1.5 mg/L (OR = 1.60; 95 % CI = 0.91–2.83) and ≥2.0 mg/L (OR = 2.46; 95 % CI = 0.95–6.34) (Table 2).

## Discussion

This propensity-matched study investigated the impact of inappropriate empirical antibiotic therapy on mortality after adjusting for potentially confounding factors that influence the receipt of inappropriate empirical antibiotic therapy in patients with HA-MRSAB in hospitals with a high prevalence of MRSA. This study found that an initial delay in the use of definitive antibiotics to which the MRSA isolates were susceptible did not necessarily prejudice the clinical outcomes of patients with HA-MRSAB and that the impetuous use of glycopeptide as empirical therapy targeting MRSA isolates should be moderated in terms of increasing antibiotic resistance.

Although initial empirical broad-spectrum antibiotic therapy, sometimes involving combination treatment, in bacteremic patients may appear to be an attractive treatment strategy before microbiological results are available, it can lead to increases in antibiotic resistance, costs, and adverse events. Studies investigating the association between inappropriate empirical antibiotic therapy and mortality among patients with bloodstream infections have reported conflicting findings. Thirteen studies have assessed the effects of empirical antibiotic therapy on mortality in study populations with MRSAB [10–16, 21, 34–36]. Seven of these study demonstrated that timely empirical therapy for MRSAB was associated with reduced mortality [10, 12–14, 21, 35, 36]. By contrast, six other studies reported an opposite result [11, 15, 16, 19, 20, 34]. Interestingly, three studies conducted in Asia reported that an initial delay in the use of definitive antibiotics to which the MRSA isolates were susceptible did not have a detrimental effect on mortality [11, 19, 20]. Paul et al. [21] speculated that the studies did not confirm an advantage to empirical vancomycin treatment, as the prevalence of vancomycin MICs >1.5 mg/L might be higher in Asia than in other locations [7, 9, 37–39].

On the contrary, a lack of agreement in the association between appropriate antibiotic therapy and mortality in bacteremic patients among the previous studies might be attributable to methodological heterogeneity. Appropriate antibiotic therapy and mortality were not consistently measured, and significant confounding variables were not always considered in the final analysis [17]. Therefore, in the present study on patients with HA-MRSAB, we obviously defined the appropriateness of antibiotic therapy in terms of its in vitro activity against the MRSA isolates, the timing and routes of administration, and the doses of the prescribed antibiotics; we also defined empirical and definitive antibiotic therapy clearly. We conducted multivariate analysis to adjust for confounding variables, including clinical severity and patients’ comorbidity at the onset of HA-MRSAB. Although the previous studies used a multivariate logistic regression analyses, residual confounding associated with the choice of appropriate or inappropriate empirical antibiotics was not fully excluded. Thus, our propensity-matched analysis calibrated all covariates that affect both the outcome and treatment assignment between patients who received appropriate and inappropriate empirical antibiotics.

In this study, there was no significant difference in mortality between the 127 pairs of propensity-matched patients who received inappropriate or appropriate empirical antibiotics. These results were comparable with the findings from a study by Kim et al*.* using a propensity score [20]*.* Among 345 patients with HA-MRSAB analyzed in our study, empirical antibiotic therapy against MRSA isolates was given to 154 (44.6 %) patients. It was previously described that infections due to gram-positive organisms do not have a rapid course, unlike those caused by gram-negative pathogens [40, 41]. In two meta-analyses of febrile neutropenic patients, there was no statistically significant difference in mortality between those who received a glycopeptide as part of the empirical regimen and those who did not receive glycopeptides [42, 43]. In addition, a large retrospective review from the National Cancer Institute suggested that the addition of vancomycin can be delayed until after four days of antibiotic monotherapy against *Pseudomonas aeruginosa* without any resulting increase in morbidity or mortality, even when a gram-positive infection was proven [44, 45].

In this study, the in-hospital mortality rate of HA-MRSAB was 33.1 %, which is comparable to the range from 29 % to 45 % reported in other studies [10, 12, 16, 36]. Within our propensity score-matched data set, three prognostic factors were associated with in-hospital mortality in patients with HA-MRSAB: severe sepsis or septic shock, Charlson’s comorbidity index at the onset of HA-MRSAB, and prior receipt of glycopeptides. These findings were similar to those of other studies [11–15, 20, 21]. In particular, prior exposure to glycopeptides was reported as an independent predictor of treatment failure for MRSAB in several studies [12, 13, 15, 20, 21]. This fact strengthens the hypothesis that the empirical use of glycopeptides should be judicious even in patients suspected to have MRSAB. In addition, a higher vancomycin MIC has been recognized as one of the predictors associated with increased mortality and treatment failure among patients with MRSAB [46, 47]. Prior exposure to glycopeptides is likely to be associated with mortality in patients with MRSAB, as recent exposure to vancomycin may lead to increased vancomycin MICs [48]. In this study, we failed to identify a significant impact of higher vancomycin MIC values on mortality in patients with HA-MRSAB, probably due to use of several antibiotics with activity against MRSAB. Vancomycin was used as a definite antibiotic in 58.3 % of the 345 patients with HA-MRSAB analyzed in the study.

There are some potential limitations in our study. First, the multivariate propensity-matched analysis still has a residual risk for confounding given the non-randomized, controlled study design. Second, it should be acknowledged that culture sampling itself might have been delayed in some cases due to difficulties in suspecting the presence of MRSAB. Third, different kinds of antibiotics used for definitive therapy against MRSA can affect the clinical outcomes. However, there was no statistical difference in the distribution of definitive antibiotics in the comparative analyses of empirical antibiotic therapy or treatment outcomes. Fourth, vancomycin MIC for MRSA isolates was determined using the thawed, stored strains in this study, which can lead to an overall decline in vancomycin MICs measured for the same strains at the time of isolation. The effects of storage on the vancomycin MIC of the isolates may be considered confounders [49]. Lastly, this study showed a lack of significant difference in the hospital mortality between patients who received inappropriate or appropriate empirical antibiotics. This may imply an inconclusive result from the limited sample size, rather than a lack of any effect of appropriate empirical antibiotic therapy.

## Conclusions

In conclusion, empirical inappropriate antibiotic therapy was not a significant predictor of mortality in patients with HA-MRSAB in hospitals with a high prevalence of MRSA. These findings suggest that it may be safe to await microbiological results and guide the use of definitive antibiotics in patients suspected of having HA-MRSAB. Although earlier proper antibiotic prescribing is usually recommended for bacteremic patients by most experts, the empirical use of glycopeptides should be guided by the presence of predictors of MRSAB as well as the risk factors associated with MRSAB-related mortality.

## Abbreviations

CR-BSI, catheter-related bloodstream infection; HA-MRSAB, healthcare-associated methicillin-resistant *Staphylococcus aureus* bacteremia; IPTW, inverse probability of treatment weighting; IQR, inter-quartile range; MICs, minimum inhibitory concentrations; MRSA, methicillin-resistant *Staphylococcus aureus*; MRSAB, methicillin-resistant *Staphylococcus aureus* bacteremia
